# Continuous response despite reduced dose of trametinib as single agent in an adolescent with a relapsed disseminated pediatric low-grade glioma *KIAA1549-BRAF* fusion positive: a case report and review of the literature

**DOI:** 10.3389/fonc.2024.1381354

**Published:** 2024-05-23

**Authors:** Serafin Castellano-Damaso, Felisa Vazquez-Gomez, Jose Luis Moreno-Carrasco, Begoña Arce, Pedro Borrego, Alvaro Lassaletta

**Affiliations:** ^1^Pediatric Neuro-Oncology Unit, Hospital Infantil Universitario Niño Jesús, Madrid, Spain; ^2^Pharmacy Department, Hospital Infantil Universitario Niño Jesús, Madrid, Spain; ^3^Radiology Department, Hospital Infantil Universitario Niño Jesús, Madrid, Spain

**Keywords:** low-grade gliomas, trametinib, MEK inhibitor, children, BRAF fusion, response

## Abstract

Dissemination in pediatric low-grade glioma may occur in about 4%–10% of patients according to retrospective cohort studies. Due to its low incidence, there is no consensus on treatment for these patients. According to the constitutional activation of the MAPK/ERK pathway in these tumors, MEK inhibitors such as trametinib have been used successfully in the relapsed setting. Skin toxicity is frequent in patients receiving trametinib, normally mild to moderate, but sometimes severe, needing to discontinue the drug, limiting the efficacy in the tumor. There is not much information in the literature regarding whether reducing the dose of trametinib is able to maintain efficacy while, at the same time, decreasing toxicity. Here, we present an adolescent, with severe skin toxicity, whose trametinib dose was reduced by 50% and efficacy on the tumor continued while skin toxicity significantly decreased.

## Introduction

Low-grade gliomas represent the most common central nervous system tumors in children (1). Although mostly localized at diagnosis, they can disseminate in up to 10% at progression or relapse (2–4). Dissemination in pediatric low-grade gliomas (pLGGs) is a phenomenon that is not fully understood (2, 5, 6), and its true incidence remains unknown due to the absence of prospective cohort studies (3). In addition, it can significantly reduce the overall survival of these patients (7, 8). According to the published case series, the frequency of dissemination in pLGG varies between 2% and 5% at diagnosis (2–4) and 2.4% and 10% at progression or relapse (2–4). Due to its low incidence, there is no universally established therapy for disseminated pLGG (3, 4, 7). As surgery is not feasible, and radiotherapy requires craniospinal radiation with many long-term side effects (7, 8), conventional chemotherapy has been the preferred treatment in disseminated pLGG (8). Recent reports state that specific molecular alterations (9–11), such as chromosome 1p deletion (11), *KIAA1549-BRAF* fusion (11), BRAF V600E mutation (12), and EGFR gene amplification (13), may contribute to cause dissemination in pLGGs. With this knowledge in mind, new targeted therapies are becoming part of its treatment (14).

MEK inhibitors have been used in the treatment of LGG in children and adolescents with promising results (14–28). However, their use in disseminated pLGG has been scarcely reported in the literature. Toxicity mainly consists of skin adverse events which can affect the quality of life of the patients sometimes needing to discontinue the drug (29). Here, we report an adolescent with a disseminated pLGG which progressed after conventional chemotherapy. Trametinib was started, resulting in a favorable tumor response but with significant toxicity. The dose was subsequently reduced by 50%, maintaining tumoral response while reducing toxicity.

## Case description

An 18-year-old non-NF1 male patient with a diagnosis of pilocytic astrocytoma of the right temporal lobe was diagnosed at the age of 3 years in 2008 at our institution, with no prior family or personal background of interest. At first, he underwent surgery on two occasions, with a partial resection at diagnosis, and another partial resection in 2010 due to tumor local progression. Due to a slow but continuous progression over time of the residual tumor and the appearance of new periventricular enhancement showed in routine magnetic resonance imaging (MRI), in December 2016, he started treatment with weekly vinblastine as per the Canadian Pediatric Brain Tumor Consortium protocol (30). Initial surgical samples were sent to the molecular biology lab for testing BRAF V600E mutation, which came up as negative. He completed 70 weeks of treatment in May 2018 with stable disease described as the best response achieved under this treatment.

After finishing chemotherapy treatment, he started to develop weekly seizures, with no signs of tumoral growth and a stable appearance of the periventricular enhancement, and started anticonvulsant levetiracetam. During one of the seizures in November 2018, he suffered a parietal bone fracture, and after noticing hydrocephalus with no signs of tumor regrowth, a ventriculoperitoneal shunt was placed. Seizures resolved.

In January 2019, a reassessment MRI showed signs of worsening leptomeningeal dissemination, both spinal and supratentorial, as well as progressive growth of the tumor remnants. Therefore, it was decided to start a second line of chemotherapy using the SIOP-LGG 2004 protocol approach (31) with vincristine and carboplatin in March 2019. Only 2 months later (May 2019), this chemotherapy regimen had to be discontinued due to significant toxicity: grade 3 peripheral sensory-motor neuropathy, grade 4 neutropenia, grade 3 thrombocytopenia, grade 3 fatigue, and grade 2 constipation. Also, he developed two new episodes of hydrocephalus and needed shunt reprogramming. Initial tumor samples were sent for further molecular studies for the purpose of testing the presence of a *KIAA1549-BRAF* fusion, as it was not tested at first due to the initial unavailability of the test. This result was positive, involving a fusion between *KIAA1549* exon 16 and *BRAF* exon 9. As a result, in July 2019, he started MEK inhibitor trametinib at standard dose (0.025 mg/kg once daily orally) (15).

**Figure 1:**
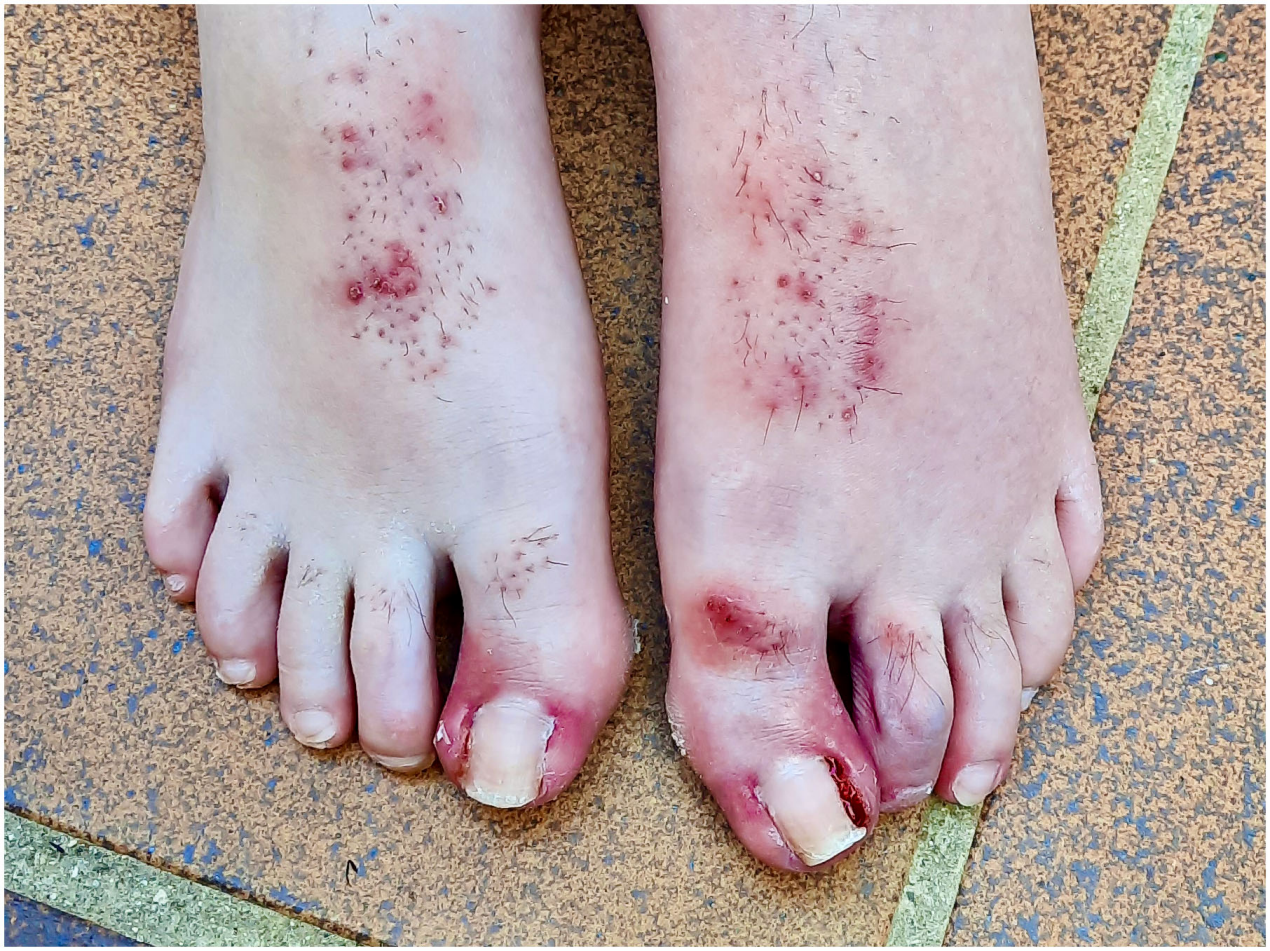
Skin toxicity in the patient showing severe paronychia in both feet.

**Figure 2:**
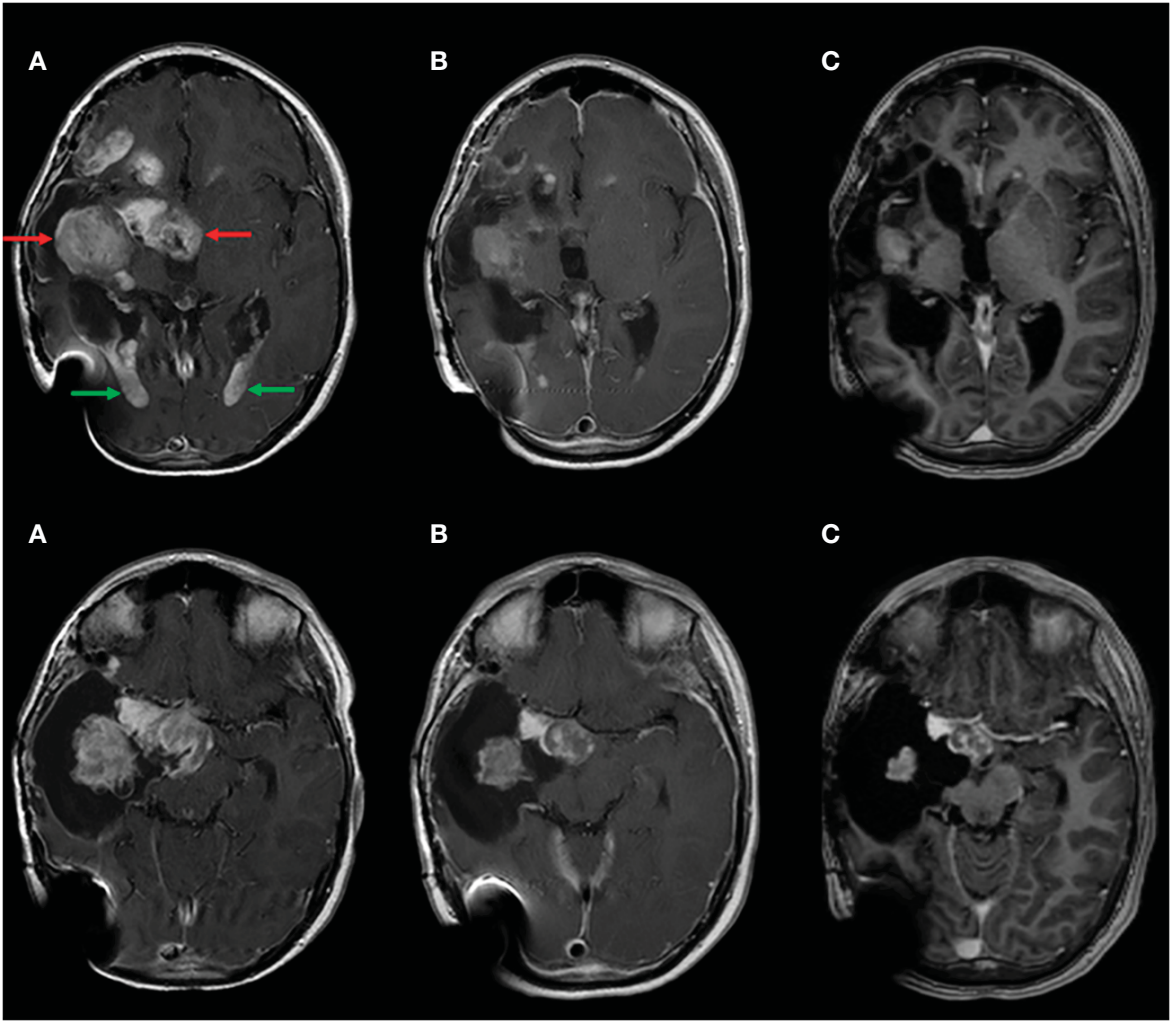
Contrast material-enhanced T1-weighted MR image shows reduction of the solid part of the main tumor (red arrows) as well as the intraventricular enhancing masses (green arrows). **(A)** Pre-treatment with trametinib; **(B)** before reducing trametinib dose; **(C)** end of treatment.

**Figure 3:**
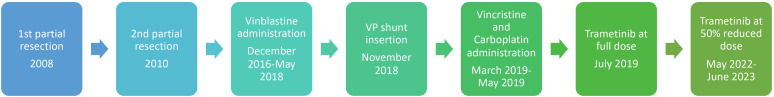
Timeline describing the treatment of the patient.

During the following 3 years of treatment, with a partial response in the follow-up MRI that was done 6 months after starting treatment, he developed grade 3 skin dermatitis, folliculitis, acneiform lesions, and paronychia (**Figure 1**), which progressively impaired the patient’s quality of life. Because of that, in May 2022, we decided to reduce the dose of trametinib to 50% of the current dose (0.0125 mg/kg once daily orally). With this new dose, all skin lesions partially resolved, and his subjective quality of life significantly improved. Despite this dose reduction, trametinib continued to reduce the size of the tumor in the 3-monthly MRIs that followed the dose reduction (**Figure 2**), maintaining a partial response even at this reduced dose. After 1 year of treatment with this reduced dose, Trametinib was finally discontinued in June 2023, after completing 4 years of therapy. So far, the patient has not developed any signs of tumor re-growth and continues to be under close follow-up at our institution (**Figure 3**).

Written informed consent to publish the case report including clinical information and images was obtained from the patient.

## Discussion

This report displays a continuous response to reduced-dose treatment with MEK-inhibitor trametinib in a patient with an initially localized pLGG, which spread over time along the neuroaxis and required a new treatment for controlling the disease.

Dissemination in pLGG may occur in about 2.4%–10% of relapses according to retrospective cohort studies (3, 4, 7). Owning to its rarity, there is no consensus about treatment for these patients (3, 4, 7). Usually, these patients have already received multiple chemotherapy lines of treatment. Craniospinal radiotherapy carries important long-term side effects, and surgery is not feasible in disseminated disease.

Almost all pLGGs have MAPK pathway alterations (1); the most frequent are the BRAF fusion *KIAA1549-BRAF* and the BRAFV600E mutation (9). Since these alterations were discovered at the beginning of the century, targeted therapy (BRAF and MEK inhibitors) has been studied in clinical trials in the relapsed/progression and new diagnosis setting (14–28). MEK inhibitors have shown efficacy in relapsed pLGG as has been reported elsewhere (16–23, 32, 33). Current knowledge in this field seems to affirm that patients with the *KIAA1549-BRAF* fusion seem to respond better to MEK inhibitors than the ones that are BRAF-fused negative (32), as in our patient´s case, whereas paradoxical reactions to BRAF inhibitors with tumor accelerated growth has been reported in these BRAF-fused patients (21, 34, 35).

There are several phase I and II clinical trials involving MEK inhibitors for treating pLGG, including selumetinib (20, 21, 26), trametinib (15–19, 23), cobimetinib (22), binimetinib (24), and mirdametinib (25). The two most researched about are selumetinib and trametinib. So far, there are multiple published results regarding selumetinib in NF1 and non-NF1 patients with pLGG. Trametinib also has been used successfully in treating progressive/relapsed low-grade gliomas in children, although achieving a complete response in monotherapy has turned out to be difficult (27, 28). Sustained responses in low-grade glioma patients have typically been achieved through the administration of these drugs; however, there are documented cases of relapse or progression following dose reduction or the discontinuation of the drug (20). In the reported literature, there have been described at least 15 patients (14, 18, 23, 32, 33) with a disseminated low-grade glioma who underwent treatment with MEK inhibitors as our patient. However, only three of them harbored the *KIAA1549-BRAF* fusion (23, 33), with an unknown BRAF status in the one reported by Selt et al, and comprising different BRAF mutations in the other eleven patients.

Toxicities are a major drawback in the treatment with MEK inhibitors. Skin toxicity is common in patients receiving trametinib (28), normally mild to moderate, but sometimes severe, needing to discontinue the drug and limiting the efficacy in the tumor. From our personal experience, side effects are usually worse in adolescents compared to younger children, although there is no strong evidence for it in the scientific literature. In our patient’s case, skin toxicity severely impaired his quality of life and obliged us to reduce trametinib’s dose as fully stopping treatment was a difficult option to take as he was responding very well to the drug. Thus, the dose reduction successfully helped to control skin toxicity while maintaining tumor response. This was in line with one of the cases published by Kondyli et al. in 2018, the only one with a detailed report in the literature. In that case, the patient was a 15-year-old female who started trametinib after progression to several lines of treatment and who also developed severe paronychia. That patient also harbored the *KIAA1549-BRAF* fusion. However, in that case, she was only followed for 6 months with no further information regarding her outcome. To our knowledge, this case report represents the longest follow-up of a disseminated low-grade glioma patient who has maintained a continuous tumoral response to trametinib despite a dose reduction to 50% due to severe adverse events.

Treatment in our patient was discontinued when the patient turned 18 years old, due to having already finished puberty. This was done based on the proposed theory that pediatric low grade gliomas stop growing after pubertal development has been completed (36). An ongoing problem in treating pLGGs with MEK and BRAF inhibitors remains regarding for how long does the treatment needs to be given. In melanoma, another MEK-pathway-driven malignant tumor, the current consensus is to maintain targeted therapy as long as a response is achieved (37), and treatment discontinuity is usually secondary to treatment toxicity or disease progression (38). Given the benign nature of pLGGs, whereas these tumors usually stop growing over time, it seems reasonable to stop treatment and do a wait-and-see approach afterward. However, the true efficacy of this management should be tested in a prospective, randomized, clinical trial. So far, our patient has not presented any kind of tumor regrowth. He is doing well and continues to be under close follow-up at our center.

This case report shows that, in very selected cases with significant toxicity to MEK inhibitors, dose reduction may improve toxicity while maintaining anti-tumor response.

The clinical characteristics of patients who may benefit from this dose-reduction approach still require further definition. Additional research is needed to determine whether pLGG patients with specific molecular alterations, such as the KIAA1549-BRAF fusion, may derive benefits from treatment with MEK inhibitors like trametinib.

## Data availability statement

The original contributions presented in the study are included in the article/**Supplementary Material**. Further inquiries can be directed to the corresponding author.

## Ethics statement

Written informed consent was obtained from the individual(s) for the publication of any potentially identifiable images or data included in this article.

## Author contributions

SC-D: Formal analysis, Data curation, Writing – review & editing, Writing – original draft, Methodology, Investigation, Conceptualization. FV-G: Supervision, Writing – review & editing, Writing – original draft. JLM-C: Writing – review & editing, Writing – original draft, Supervision. BA: Validation, Investigation, Writing – original draft, Supervision. PB: Software, Writing – original draft, Validation, Supervision. AL: Writing – review & editing, Methodology, Investigation, Funding acquisition, Conceptualization, Writing – original draft, Validation, Supervision.
